# Supplementary material for: Reassessing the winner-loser gap in satisfaction with democracy
Source: PLoS One. 2024 Dec 17;19(12):e0314967. doi: 10.1371/journal.pone.0314967 (PMC11651613; doi:10.1371/journal.pone.0314967)
Supplement: S1 Appendix — (PDF) [file pone.0314967.s001.pdf]

Supporting information

Appendix A: Operationalizations and descriptive statistics

Table S-A1 below presents the party choices available to respondents in the three surveys, along with the coding that indicates whether the corresponding party choice classifies the respondent's status as either a winner or a loser for the analysis of their satisfaction with democracy.

Table S-A1. Party choices available to respondents and their corresponding winner/loser coding.

**Norway 1997**	
Survey response for party choice	Coded as
01. Red Electoral Alliance	Loser
02. Socialist Left Party	Loser
03. Labor Party	Loser
04. Liberal Party	Winner
05. Christian People's Party	Winner
06. Center Party	Winner
08. Conservative Party	Loser
09. Progress Party	Loser
10. Other Parties	Loser

Table S-A1. (*cont.*) **Party choices available to respondents and their corresponding winner/loser coding.**

**Iceland 2017**	
Survey response for party choice	Coded as
1 Social Democratic Alliance	Loser
2 Progressive Party	Winner
3 Independence Party	Winner
4 Left-Green Movement	Winner
5 Bright Future	Loser
6 Pirate Party	Loser
7 Dawn	Loser
8 Reform Party	Loser
9 People's Front of Iceland	Loser
10 Centre Party	Loser
11 People's Party	Loser
89 Another party	Loser

**Netherlands 2012**	
Survey response for party choice	Coded as
1 Party for Freedom and Democracy	Winner
2 Labour Party	Winner
3 PVV (List Wilders)	Loser
4 Christian Democratic Party	Loser
5 Socialistic Party	Loser
6 Democrats '66	Loser
7 Green Left	Loser
8 Christian Union	Loser
9 Social Reformed Party	Loser
10 Party for the Animals	Loser
11 Pirate Party	Loser
12 50Plus	Loser
13 Other	Loser

Fig S-A1 shows the distribution of the surveys around the cutoff dates for each election. The number of respondents varies from 945 for the Netherlands in 2012, 1470 for Iceland 2017, to 1703 for Norway 1997. There are sufficient respondents either side of the cutoff. The number of winners and losers is quite similar in the Netherlands and Iceland. For Norway, however, the number of electoral losers who were interviewed is larger than the number of electoral winners.

Fig S-A1. The timeline of the responses and the electoral status by election.

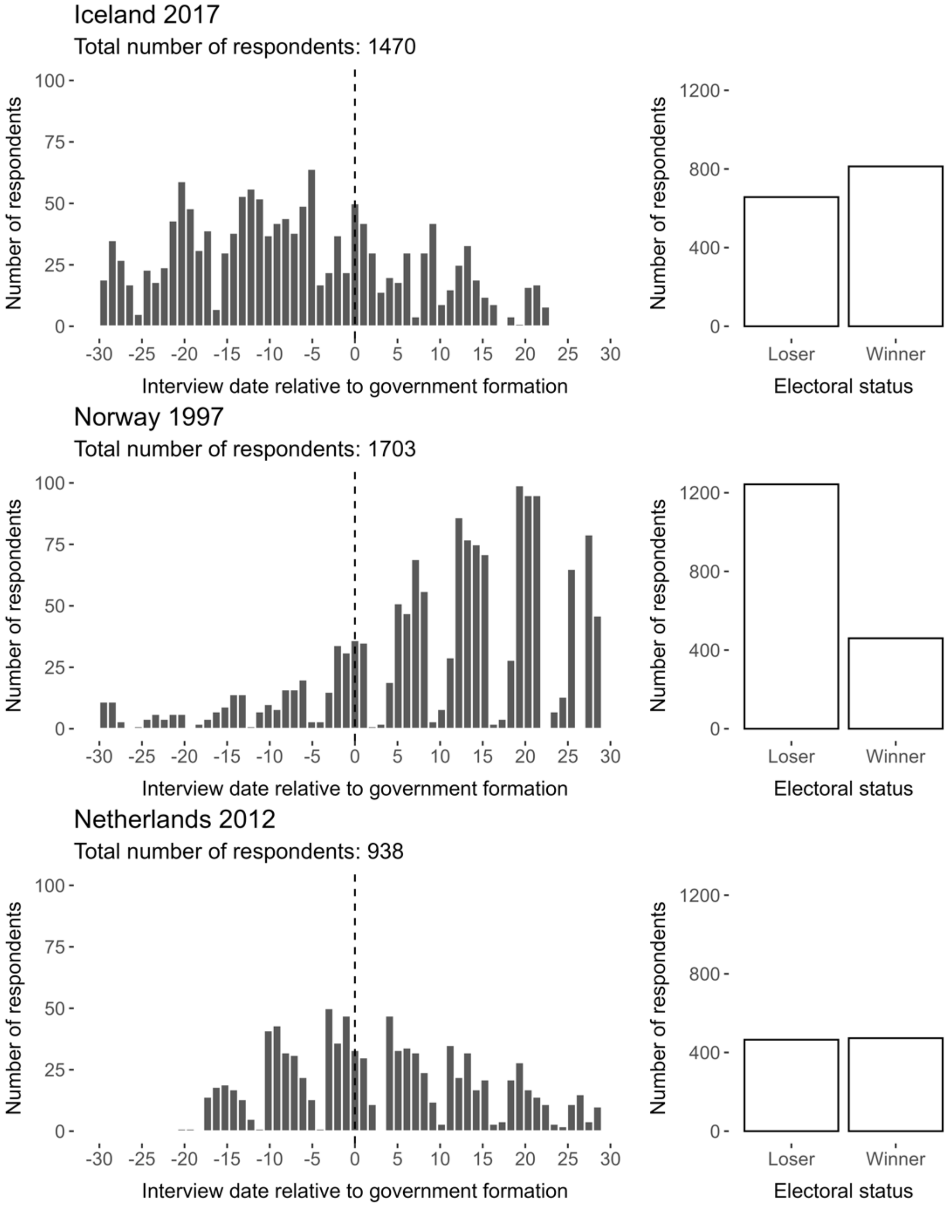

Appendix B: Balance statistics

One of the assumptions of the regression discontinuity design that respondents interviewed before the cutoff date and after the cutoff date are similar. Therefore, within the algorithmically chosen bandwidth, we expect that the proportion of respondents who are electoral winners/losers should be similar before and after the cutoff date. Furthermore, respondents should also be similar with respect to important socio-economic markers and political attitudes such as gender, age, income, education, ideology, and their geographic regions. We examine the balance on these variables. Table S-B1 focuses on the Netherlands (2012) and shows that for most makers there are neither substantial nor systematic differences between those who were interviewed before their electoral status became known and those who were interviewed after their electoral status became known. There are two exceptions. Respondents interviewed after the electoral status became known are on average about 3 years younger (52 versus 55) than those interviewed before. In addition, there are six percentage points fewer respondents from the South of the Netherlands regions. Overall, it is encouraging that the proportions of respondents who were electoral winners/losers did not change systematically when the electoral status became common knowledge.

Table S-B1. Balance statistics for the Netherlands 2012.

Variable	Mean before	Mean after	Mean diff.	p-value
Electoral Status	0.53	0.49	-0.03	0.28
Female	0.51	0.53	-0.01	0.71
Age	55.28	52.11	-3.16	0.00
Income	5.95	6.26	0.31	0.09
Income NA	0.11	0.12	0.01	0.51
Education 1	0.09	0.06	-0.02	0.15
Education 2	0.31	0.31	0.00	0.97
Education 3	0.21	0.25	0.04	0.14
Education 4	0.01	0.02	0.01	0.24
Education 5	0.38	0.36	-0.02	0.42
Left-right	5.22	5.31	0.09	0.50
District 1	0.10	0.12	0.02	0.24
District 2	0.21	0.21	-0.01	0.79
District 3	0.44	0.48	0.04	0.18
District 4	0.25	0.19	-0.06	0.02

Balance statistics are estimated within the wider robust bandwidths as the corresponding RDDs (see S-C1 Table). The variable *Income* measures the household income in 10 categories. We have treated this variable as continuous. For the Netherlands sample, household income is also the only variable with a larger amount of missingness (49 in the electoral winners sample and 60 in the electoral losers sample). For this reason, we include an indicator of whether the household income variable was missing *Income NA* in the balance statistics. Missingness was balanced both before and after the cutoff date. *Education* is measured in five separate categories, *Left-right* asks the respondents to self-assess their ideology on an eleven-point scale. The variable *District 1* contains respondents from the North Netherlands region. More specifically: Groningen, Oost-Groningen, and Delfzijl en omgeving. *District 2* contains respondents from East Netherlands: Overijssel, Gelderland, and Flevoland. *District 3* includes respondents from the West of Netherlands districts: Utrecht, Noord-Holland, Zuid-Holland, and Zeeland. *District 4* includes respondents from the South of Netherlands regions: Noord-Brabant and Limburg.

Table S-B2 shows the balance test for Norway (1997). Overall, the Norwegian sample used in the RDD, within the bandwidth of 23/16 is well balanced. There are neither substantial nor systematic differences in the samples taken before and after the cutoff. The Norway samples are well balanced overall. However, respondents surveyed after the electoral status became common knowledge are on average 8 years younger than those

surveyed before. Furthermore, the share of respondents from income category one is 4 percentage points larger after the cutoff. The Iceland samples are well balanced overall, as shown by Table B3. However, respondents surveyed after the electoral status became common knowledge are on average 8 years younger than those surveyed before. Furthermore, the share of respondents from income category one is 4 percentage points larger after the cutoff.

Table S-B2. Balance statistics for Norway 1997.

Variable	Mean before	Mean after	Mean diff.	p-value
Electoral Status	0.27	0.26	-0.02	0.63
Female	0.49	0.47	-0.02	0.65
Age	43.17	43.35	0.18	0.88
Income 1	0.19	0.18	-0.01	0.72
Income 2	0.20	0.15	-0.05	0.07
Income 3	0.12	0.17	0.05	0.09
Income 4	0.19	0.24	0.05	0.11
Income 5	0.25	0.20	-0.04	0.19
Education 3	0.17	0.20	0.03	0.31
Education 4	0.23	0.20	-0.03	0.28
Education 5	0.33	0.34	0.01	0.81
Education 8	0.26	0.26	0.00	0.97
Left-right	5.35	5.10	-.25	0.14
District 1	0.36	0.31	-0.04	0.25
District 2	0.18	0.18	0.00	0.94
District 3	0.05	0.05	0.00	0.89
District 4	0.21	0.25	0.04	0.24
District 5	0.09	0.09	0.00	0.95
District 6	0.11	0.11	0.00	0.90

Balance statistics are estimated within the wider robust bandwidths as the corresponding RDDs (see S-C2 Table). The education variable contains nine categories. The only categories with a substantial number of respondents are category three “primary completed”; category four “incomplete secondary”; category five “secondary completed”; category eight “university undergraduate degree completed.” Two observations are missing. The income variable in Norway is measured in quintiles with nine observations missing. The region variables in Norway are District 1: “Oslofjord”; District 2: “Inner East of Norway”; District 3: “Southern Norway”; District 4: “Western Norway”; District 5: “Trandelag”; District 6: “Northern Norway.”

Table S-B3. Balance statistics for Iceland 2017.

Variable	Mean before	Mean after	Mean diff.	p-value
Electoral Status	0.58	0.58	0.00	0.99
Female	0.48	0.50	0.02	0.63
Age	51.79	43.80	-7.99	0.00
Income 1	0.13	0.18	0.05	0.04
Income 2	0.15	0.13	-0.02	0.47
Income 3	0.17	0.15	-0.02	0.49
Income 4	0.14	0.13	-0.01	0.65
Income 5	0.18	0.15	-0.04	0.14
Income NA	0.23	0.26	0.03	0.27
Education 3	0.23	0.26	0.03	0.27
Education 4	0.23	0.26	0.03	0.27
Education 5	0.23	0.26	0.03	0.27
Education 6	0.23	0.26	0.03	0.27
Education 7	0.23	0.26	0.03	0.27
Education 8	0.23	0.26	0.03	0.27
Left-right	5.37	5.37	0.00	0.99
District 1	0.14	0.12	-0.02	0.35
District 2	0.11	0.08	-0.03	0.13
District 4	0.13	0.14	0.02	0.48
District 5	0.27	0.27	0.00	0.91
District 6	0.17	0.19	0.02	0.51
District 7	0.17	0.20	0.02	0.35

Balance statistics are estimated within wider robust bandwidths as the corresponding RDDs (see S-C3 Table). Income is measured in quintiles. However, there is more missingness on income (23%). Education is measured in eight categories, however, there are just three respondents from the two lowest categories in the sample overall. Categories one and two, are therefore, omitted. Among winners and losers, most respondents cluster in the highest education category (university undergraduate degree completed) with few respondents in the other categories. The Iceland samples contain respondents from six districts: Northeast constituency, South constituency, Southwest constituency, Reykjavik south, and Reykjavik north. There are no respondents from district 2 which is the Northwest constituency.

Appendix C: Regression tables

The results for Netherlands 2012 election are insignificant and relatively stable across the different methods. For electoral winners, the results are also well powered for effect sizes of 0.4 or larger for both conventional and robust estimates with power larger than 0.8. Similarly, for electoral losers the design is well powered for effect sizes of 0.5 or larger.1

Table S-C1. RD Estimates for the Netherlands 2012.

	**Electoral Winners**			**Electoral Losers**		
Method	Coefficient	95% CI		Coefficient	95% CI	
Conventional	-0.03	-0.27	0.20	-0.06	-0.33	0.21
Bias-Corrected	-0.10	-0.34	0.13	-0.06	-0.33	0.21
Robust	-0.10	-0.39	0.19	-0.06	-0.38	0.25
N	687			664		
BW est. (std.)	14/23			12/37		
BW est. (robust)	20/66			17/108		

Estimated with the *rdrobust* package (v2.2) in R. Reported methods are 1) conventional RD estimates with conventional standard errors, 2) bias-corrected estimates with conventional standard errors, 3) bias corrected estimates with bias-corrected standard errors.

In the Norway 1997 election, the results are insignificant for electoral winners and electoral losers independent of the method and the associated bandwidth. As argued in footnote 7, when looking at one single election, power may be less of an issue if one accepts

[1] Given that we are investigating a single election, power may arguably be irrelevant if we do not regard the Netherlands 2012 election as a sample.

that the particular case is not a sample. Otherwise, the Norwegian case is such that we need an effect size of 0.7 to achieve power greater than 0.8 with the robust approach for both electoral winners and electoral losers.

Table S-C2. RD Estimates for Norway 1997.

	**Electoral Winners**			**Electoral Losers**		
Method	Coefficient	95% CI		Coefficient	95% CI	
Conventional	-0.21	-0.86	0.45	0.07	-0.51	0.64
Bias-Corrected	-0.22	-0.87	0.44	0.04	-0.54	0.62
Robust	-0.22	-0.92	0.49	0.04	-0.59	0.68
N	471			1247		
BW est. (std.)	9/6			6/6		
BW est. (robust)	23/16			17/14		

Estimated with the *rdrobust* package (v2.2) in R. Reported methods are 1) conventional RD estimates with conventional standard errors, 2) bias-corrected estimates with conventional standard errors, 3) bias corrected estimates with bias-corrected standard errors.

The results for the Iceland 2017 election are also insignificant for both electoral winners and electoral losers independent of the method and associated bandwidth. For the robust estimates to be well powered, we require effect sizes of 0.7 for electoral losers and 0.9 for electoral winners. The conventional approach is well powered, however, at a much smaller effect size of 0.16. Given that the estimates do not differ too much between the approaches we are more confident in the null findings.

Table S-C3. RD Estimates for Iceland 2017.

	Electoral Winners			Electoral Losers		
Method	Coefficient	95% CI		Coefficient	95% CI	
Conventional	-0.17	-1.02	0.69	0.41	-0.66	1.48
Bias-Corrected	-0.19	-1.04	0.66	0.38	-0.69	1.50
Robust	-0.19	-1.09	0.71	0.38	-0.75	1.51
N	879			717		
BW est. (std.)	4/8			5/7		
BW est. (robust)	13/8			14/20		

Estimated with the *rdrobust* package (v2.2) in R. Reported methods are 1) conventional RD estimates with conventional standard errors, 2) bias-corrected estimates with conventional standard errors, 3) bias corrected estimates with bias-corrected standard errors.

Appendix D: Robustness checks

In the main manuscript, Fig 5 presents RDD's with varying cutoffs for the pooled sample including all three elections. Here, Fig S-D1 shows the results for each election separately. The same pattern as in the pooled model holds. The RDD effects are mostly insignificant. In the few cases where we find an effect that would be consistent with our expectation, the dates at which the effect occurs are not consistent across electoral winners and losers. For example, for the Iceland election, we find a positive effect five days before our cutoff. At five days before the actual cutoff (as well as the days around it) we do not observe a negative effect for the electoral losers. On balance, the plots are consistent with a null result and the expected number of false positives.

Fig S-D1. Separate election RDD's with varying cutoffs. Robust Doughnut RDD estimates with varying cutoff dates on the x-axis and 95% confidence intervals.

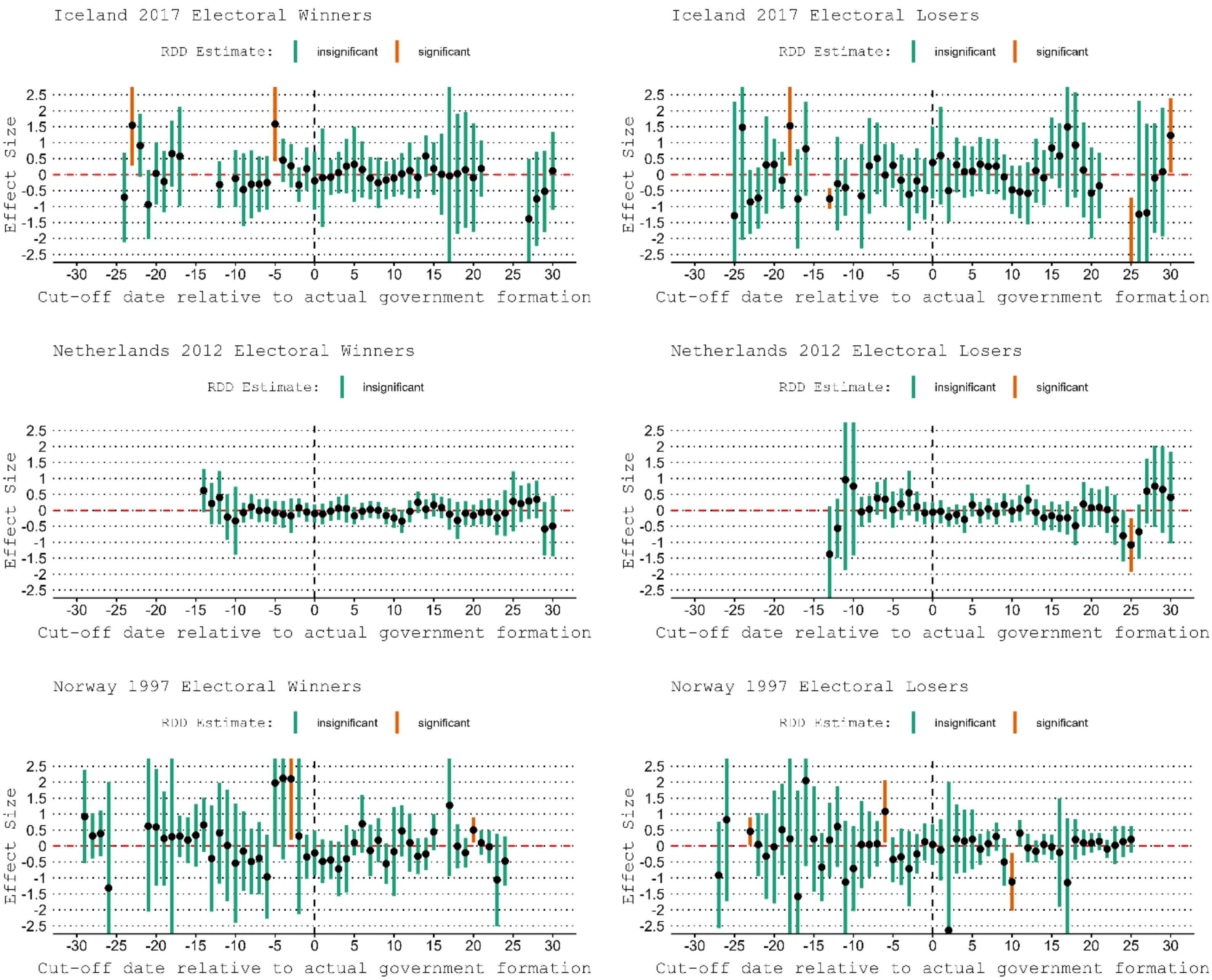

In our analysis, we excluded those who abstained or cast an invalid ballot. It can be argued that abstainers should be included into the category of electoral losers. Table S-D1 below illustrates our findings when we include abstainers into the analysis as electoral losers. Our findings remain the same, electoral losers are not less satisfied with the way democracy works after the electoral status has been revealed.

Table S-D1. Pooled Elections RD Estimates Including Abstainers as Electoral Losers.

Electoral Losers<br>Method	Coefficient	95% CI	
Conventional	0.12	-0.17	0.42
Bias-Corrected	0.14	-0.15	0.44
Robust	0.14	-0.16	0.45
N	1972		
BW est. (std.)	7/12		
BW est. (robust)	22/52		

Estimated with the *rdrobust* package (v2.2) in R. Reported methods are 1) conventional RD estimates with conventional standard errors, 2) bias-corrected estimates with conventional standard errors, 3) bias corrected estimates with bias-corrected standard errors.
