# Supplementary material for: Reassessing the winner-loser gap in satisfaction with democracy
Source: PLoS One. 2024 Dec 17;19(12):e0314967. doi: 10.1371/journal.pone.0314967 (PMC11651613; doi:10.1371/journal.pone.0314967)
Supplement: Attachment — Submitted filename: PONE-D-24-06817 review.docx [file pone.0314967.s002.docx]

**General assessment:**

The paper addresses whether the winner-loser status explains satisfaction with democracy. By applying a regression discontinuity design to data from three elections—Norway 1997, the Netherlands 2010, and Iceland 2017—the authors’ main finding do not confirm the theoretical expectations. In other words, winners do not show higher levels of satisfaction with democracy, and losers are not less satisfied with democracy. Although limited to three cases, the causal inference design allows for their main claim: the winner-loser gap does not explain satisfaction with democracy, challenging decades of knowledge regarding the topic.

Whereas the paper’s main strength is establishing a causal relation between the winner-loser status and satisfaction with democracy, its weakness might be the impossibility of external validity. On the one hand, the authors address the question through a careful research design that considers the assumptions of RDD and assigns robustness to their findings through standard tests. On the other hand, it is hard to claim that such findings are valid for other cases rather than those analyzed.

**Major issues:**

The paper has no significant issues.

**Minor issues:**

The way in which the authors present their conclusion could be done more carefully. Both in the introduction (lines 97-98) and conclusion (lines 303-306 and 310-311), the authors state their conclusion in a way that might make it seem as if previous literature is misleading - which is not accurate. Although prior tests usually rely on probabilistic research designs (therefore, establishing a correlation between variables) using the winner-loser gap as one of the predictors of various dependent variables outcomes (satisfaction with democracy, trust in political institutions, etc.), the study of three cases alone does not falsify all previous research. The authors are not explicitly arguing this, but the way that they present it – that “party government status does not seem to explain why winners tend to be more satisfied with democracy than losers” – sounds like the findings are generalizable.

Approaching the issue through causal inference techniques is undoubtedly an important step. However, do the conclusions apply beyond these cases? Is there external validity? The findings bring some theoretical implications because theoretical expectations are not confirmed. Yet, the authors should be more cautious in presenting their argument, clarifying that the conclusion only applies to the cases under scrutiny.

The minor issue mentioned above is the most crucial one. Below, I point to a few less substantial but important things to address.

The article needs to be revised for typos and misplaced sentences. A few sentences appear in the main text but should be footnotes or endnotes (lines 197-200 and 212-214, for instance).

Regarding how authors present their findings, I suggest bringing Table 4 from the Appendix to the text.

Figure 4 presents the results. I believe the authors are plotting polynomial regressions, which is a valid method. However, I wonder if a simple linear fit would be better for visualizing continuity assumptions. Although the authors do not confirm expectations, they find significant results contrary to the expectations for winners. Therefore, the running variable discontinuously jumps at the cutoff—even if it jumps downwards. Would the jump occur in the absence of the treatment?

Still related to Figure 4, the authors could centralize the cutoff on the plot. The X-axis should have the same number of days on each side of the cutoff.

Overall, this is a fascinating and promising paper that applies cutting-edge methods to a classic question in public opinion studies. Therefore, my recommendation is to revise minor issues and resubmit.
