# Supplementary material for: Reassessing the winner-loser gap in satisfaction with democracy
Source: PLoS One. 2024 Dec 17;19(12):e0314967. doi: 10.1371/journal.pone.0314967 (PMC11651613; doi:10.1371/journal.pone.0314967)
Supplement: Attachment — Submitted filename: memo_PLOS_final.docx [file pone.0314967.s003.docx]

***PLOS ONE***

**“*Reassessing the Winner-Loser Gap in Satisfaction with Democracy*”**

**PONE-D-24-06817**

**Comments from the Editor**

This manuscript employs a regression discontinuity design (RDD) to assess the impact of political winner/loser status on democratic satisfaction. The causal identification strategy relies on pinpointing public opinion surveys conducted concurrently with the announcement of government formation outcomes. The sample comprises three elections from Western Europe, specifically in the Netherlands, Norway, and Iceland. Nevertheless, several concerns raised by the referees must be addressed in a revised version of the manuscript.

It is necessary to review the mistaken logic of the Balance Tests presented in the appendix, which are crucial for the validity of the research design. It is necessary to discuss the limits of the external validity of the test with the analysis of only 3 elections in countries that have many similar characteristics, which are far removed from the reality of most contemporary democracies. Thus, despite the interest of the findings, which need to be validated with more tests, their conclusions need to be appropriate to the limits of the research design adopted. We kindly ask the authors to pay attention to all the reviewers' comments.

***We are grateful for the opportunity to submit a revised iteration of our manuscript and we thank the editor and reviewers for their careful reading and comments. In this memo, we detail how we have revised our manuscript. The reviewers’ comments are copied in this note, and they are followed by our responses. For clarity, the reviewers' comments are in blue font, followed by our responses in black font.

**Reviewer's Responses to Questions**

1. Is the manuscript technically sound, and do the data support the conclusions?

The manuscript must describe a technically sound piece of scientific research with data that supports the conclusions. Experiments must have been conducted rigorously, with appropriate controls, replication, and sample sizes. The conclusions must be drawn appropriately based on the data presented.

Reviewer #1: Yes

Reviewer #2: Partly

Reviewer #3: Yes

2. Has the statistical analysis been performed appropriately and rigorously?

Reviewer #1: Yes

Reviewer #2: Yes

Reviewer #3: Yes

3. Have the authors made all data underlying the findings in their manuscript fully available?

The [PLOS Data policy](https://can01.safelinks.protection.outlook.com/?url=http%3A%2F%2Fwww.plosone.org%2Fstatic%2Fpolicies.action%23sharing&data=05%7C02%7Cthomas.gareaupaquette%40mail.mcgill.ca%7C3f1aa9bc95dc40ec494908dc74ed3f7b%7Ccd31967152e74a68afa9fcf8f89f09ea%7C0%7C0%7C638513810888541625%7CUnknown%7CTWFpbGZsb3d8eyJWIjoiMC4wLjAwMDAiLCJQIjoiV2luMzIiLCJBTiI6Ik1haWwiLCJXVCI6Mn0%3D%7C0%7C%7C%7C&sdata=09PtbD%2FYe2au8EMpwZhphPYwHXA90VZc9L1dDZFDzJE%3D&reserved=0) requires authors to make all data underlying the findings described in their manuscript fully available without restriction, with rare exception (please refer to the Data Availability Statement in the manuscript PDF file). The data should be provided as part of the manuscript or its supporting information, or deposited to a public repository. For example, in addition to summary statistics, the data points behind means, medians and variance measures should be available. If there are restrictions on publicly sharing data—e.g. participant privacy or use of data from a third party—those must be specified.

Reviewer #1: Yes

Reviewer #2: Yes

Reviewer #3: Yes

4. Is the manuscript presented in an intelligible fashion and written in standard English?

PLOS ONE does not copyedit accepted manuscripts, so the language in submitted articles must be clear, correct, and unambiguous. Any typographical or grammatical errors should be corrected at revision, so please note any specific errors here.

Reviewer #1: Yes

Reviewer #2: Yes

Reviewer #3: Yes

**Reviewer #1**

This manuscript uses a regression discontinuity design (RDD) to estimate the effect of political winner/loser status on satisfaction in democracy. The causal identification strategy is based on identifying public opinion surveys that took place while the outcomes of government formation processes were announced. The sample includes three elections that took place in Western Europe (the Netherlands, Norway and Iceland).

As the authors rightly note, the gap in satisfaction with democracy and other attitudes between political winners and losers is one of the best documented and robust findings of empirical pollical science. Tracing the mechanisms that account for this gap is an important problem in this literature. In principle, using RDD based on the quasi-random assignment of survey respondents to winner/loser status based on the timing of announcing the outcomes of government formation processes can be a valid strategy. The empirical basis of this paper, however, is quite limited with 3 elections that all took place in old, well-established democracies with proportional representation – all factors that attenuate the effects of electoral winning and losing. Moreover, the government formation process in the Netherlands in particular ended in an outcome that makes it very hard to assign winners and losers, even in the narrower sense of government participation, since the PVV ended up as a party supporting the government but not formally being part of it. (In addition, CDA became part of the government but suffered a significant loss at the election and lost the prime-ministerial position, which makes it a very unusual type of ‘winner’).

So, while the result that using this particular design on these three elections, one cannot detect an effect of political winning on satisfaction with democracy is interesting and valid (even taking into account some questions on the methodology that I have below), I think the relevance of the result is limited based on the empirical scope of the analysis.

***We thank Reviewer 1 for their careful review.

First, we agree that the external validity of our findings is limited by the three countries included, which are not representative of democracies around the world. Thanks to R1’s comment, we now better acknowledge this limitation:

-In the introduction, we clarified “in well-established democracies” when referring to our main conclusion.

-In the method section, we added the following:

“The external validity of our case studies is constrained by the fact that the three countries included are not representative of most democracies in the world. Indeed, they are well established democracies using proportional representation, which has been shown to reduce the winner-loser gap [34]. That said, even democracies of highest quality still display a winner-loser gap in citizens’ political support [35]. Indeed, Erhart mentions that “The winner-loser gap persists even in a consolidated, well-functioning consensus democracy like the Netherlands.” [26] However, given the characteristics of the countries included in our samples, we do not extrapolate our findings to democracies in general, but rather to the three case studies that we could include.”

-We conclude the paper by calling for further research that would improve the external validity of our findings:

“However, our findings are limited to a set of well-established democracy using proportional representation. Further research should aim to expand cases study outside well-established democracies to assess the external validity of our findings, and should also aim at testing contextual effects related to whether the winners supported a major or junior coalition member of the government, as well as levels of polarisation of a party systems.”

Second, we agree that the government formation process in the Netherlands was quite messy since the PVV ended up as a party supporting the government without being formally part of it. Indeed, the PVV supported the government in parliament, but did not have any of its member as part of the cabinet. This issue made us realize that we should have been clearer regarding our definition of winner and loser, and their operationalizations in the selected cases. Therefore, and thanks to R1’s comment, the manuscript now explicitly mentions which parties formed the respective governments and that their supporters are coded as winners:

“The winner-loser status was coded based on respondents’ self-reported vote choice. After the 1997 elections in Norway, the government was formed by the Liberal Party, the Christian People’s Party, and the Center Party. Hence, their self-reported voters are coded as winners, while all other responses referring to Norwegian political parties are coded as losers. In Iceland, following the 2017 elections, the post-election government was formed by the Progressive Party, the Independence Party, and the Left-Green Movement. Thus, their voters are considered winners in our analysis, while respondents who chose other parties are coded as losers. In the Netherlands, after the 2012 elections, a minority cabinet was formed by the Party for Freedom and Democracy and the Labour Party, with support from the PVV (List Wilders). Consistent with Erhard [26], we coded only the voters of the first two parties as winners, as these were the only parties with representatives nominated into the government. Since the PVV did not have a government portfolio and did not directly participate in the government, their voters were coded as losers, along with voters of other opposition parties.”

In addition, the paragraph now informs the reader that the full list of party choices offered to respondents in the selected surveys, along with the corresponding winner/loser coding, has been added to Appendix A (see S-A1 Table). We apologize for any confusion and hope that these revisions will help clarify our coding choices.

With regards to the conceptual definition, we now clearly define electoral winners and losers, whose electoral status is based on whether the party they supported is included or excluded from the government. We also define what being included (or excluded) in the government means. That is, to be considered as included in the government, a party needs to have at least one cabinet member from its party. Hence, with respect to the Dutch case, the PVV does not fall into the “winner” category as they did not have any representation in cabinet. This is consistent with Erhart (2023: 210) who studies the winner-loser gap in the Netherlands and mentions: “I treat the PVV as not part of the Rutte [I] government, since they only supported the government by confidence and supply, but held no cabinet seats.”

We clarified our approach in the revised manuscript and also gave the very example of a party supporting a government in parliament without having any representation in the cabinet. See these two paragraphs from the literature review and the method section:

[From the literature review section]: “In contrast to the uncertainty regarding the mechanism(s), the literature is much clearer when it comes to defining the winners and losers. Being a “winner” or a “loser” taps onto a party’s performance. Many measures can be used to capture party performance. First, one can focus on vote share, seat share and/or participation in government [17]. Second, these three sets of indicators can be either based on a given election (absolute performance) or compared to the party’s previous electoral results (relative performance) [18]. However, the focus has been put on whether a party is included or excluded from a government, that is, whether one’s party is in power or not [1]. Winners are thus voters who supported a party that ends up with cabinet members, while losers are supporters of parties ending up in the opposition. Stiers, Daoust, and Blais [19] examine citizens’ own perceptions of having won or lost an election and show that whether a voter’s party is included in government is the most important determinant of whether the voter views themselves as a winner. In other words, being in government is winning. That said, other measures like the relative vote share between the previous election and a given election is also useful, and the media’s coverage of the electoral outcomes also shape which parties are depicted as winners or losers [20,21]. At the end of the day, however, we follow the conventional wisdom, and in particular the pioneer work of Anderson et al. [1], by defining winners and losers based on whether the party they supported is included or excluded from the government.”

[From the method section]: “The winner-loser status was coded based on respondents’ self-reported vote choice. After the 1997 elections in Norway, the government was formed by the Liberal Party, the Christian People’s Party, and the Center Party. Hence, their self-reported voters are coded as winners, while all other responses referring to Norwegian political parties are coded as losers. In Iceland, following the 2017 elections, the post-election government was formed by the Progressive Party, the Independence Party, and the Left-Green Movement. Thus, their voters are considered winners in our analysis, while respondents who chose other parties are coded as losers. In the Netherlands, after the 2012 elections, a minority cabinet was formed by the Party for Freedom and Democracy and the Labour Party, with support from the PVV (List Wilders). Consistent with Erhard [26], we coded only the voters of the first two parties as winners, as these were the only parties with representatives nominated into the government. Since the PVV did not have a government portfolio and did not directly participate in the government, their voters were coded as losers, along with voters of other opposition parties. In all three cases, abstainers were excluded from the analysis, but we should note that merging them with losers do not alter our conclusions, see Table D1 in Appendix D. The full list of party choices and their corresponding coding is provided in S-A1 Table in Appendix A.

On the use of recall question regarding respondents’ vote choice and their validity, see Fournier et al. [38] and Dassonneville and Hooghe [39]. We followed the conventional wisdom regarding the notions of winning and losing (as reviewed previously) by conceiving as winners voters who cast their ballot for a party included in the governing coalition after the election. Losers are those who supported an opposition party. To be considered as “included” in government, a party needs to have at least one elected representative in the cabinet. Hence, parties who support a government in parliament (whether informally or formally by following, for example, a confidence and supply agreement) but do not have any representation through a cabinet member are considered as not being part of the government. This approach assumes that being in or out of the government is key [1]. This assumption is, we believe, reasonable (which is evidenced by the scholarship using this approach), and also echoes Daoust, Plescia and Blais’ [16] findings on citizens’ public opinion: “Yes, having voted for a party making it to the government produces a feeling of winning, and yes, it leads to greater satisfaction with the state of democracy.”

Moreover, we also acknowledge that it is not the only measure that can be used to measure party performance—see our response to the first “additional point” made by R1.

**Balance tests**

A crucial assumption underlying the research design of the paper is that the assignment of survey respondents before and after the announcement of the new government is as-random. That would be the case if the survey recruitment procedure was truly random and there was no differential non-response to the survey that was related the winner/loser status. To validate this assumption, the authors present balance tests in the Appendix. These balance tests, however, examine whether the populations of winners and losers are different before and after the cut-off, which is not the question of interest. The question of interest is whether the samples of respondents interviewed before and after the cut-off point differ across a range of possibly-relevant characterises. This would not be the case, for example, if stratified random sampling was employed by the survey companies collecting the data, where some regions of the country interviewed before others. The design assumption would be violated as well if political losers had a higher propensity to decline an interview on a (political) survey after the announcement of the elections (or to reveal who they voted for).

***This is a very good point.

Thanks to the reviewer’s comment, we now present revised balance tests in Appendix B. That is, we present balance tests for the marginal distributions of the following relevant characteristics: 1) proportion of electoral winners; 2) gender; 3) age; 4) income; 5) education levels; 6) self-reported left-right ideology; 7) regions. Overall, the new balance tests are very reassuring.

In the Netherlands, we compare 15 averages before and after the cut-off, and only 2/15 (for age and Western Netherlands) are imbalanced (*p*<.05). In Norway, none of the 19 averages are different before and after the cut-off. In Iceland, 1/22 difference is statistically significant (for age).

Overall, there are no systematic differences in the electoral status and the regional proportions (with one exception in the Netherlands).

See the revised Appendix B for details.

Another crucial assumption on which the design rests is that the news about the government formation could not have been anticipated and internalized before the announcement. I think this is hard to justify in some contexts, where potential coalition partners strike a deal on general participation in government together before hammering out over months the details of the coalition agreement (such as the Netherlands). In such cases, one would not indeed expect that the announcement of the government formation itself changes much for anyone.

***R1 is right that this is one of the assumptions of the paper. As from the manuscript, “Our research design requires post-electoral circumstances in which the composition of the incoming government was highly uncertain.” To make sure that it was the case, we began with 389 cases and completed three rounds of exclusions. See in particular the third step:

“In the third step, we conducted a qualitative inquiry into the remaining elections by reviewing national election reports and news media to determine the level of uncertainty related to the government formation process. At the end of the day, three elections that satisfied our scope conditions were: Norway (1997) and Iceland (2017) from the CSES and the Netherlands (2010) from the ESS. For descriptive statistics relevant to the winner-loser gap and the timing of survey interviews, refer to S-A1 Fig in Appendix A. For additional information on the political context surrounding the three cases, see Harđarson and Önnudóttir [29], Van Holsteyn [30], Madeley [31], Pellikaan, de Lange, and van der Meer [32], and Valen [33] for electoral reports.”

Overall, we believe that the three case studies meet the criteria to be included in our research.

One might be less convinced about one of the three cases, but the findings from individual elections are consistent with our general conclusion, should be reassuring for a reader who is less convinced, for example, that the Netherlands should be included compared to the other cases.

On a side-note, to compare how their estimates differ from panel-data based estimations of the winner-loser gap, the authors could use the LISS survey in the Netherlands and estimate the same effect with that alternative data source and design, in order to be able to pinpoint more precisely where the difference with existing findings comes from.

***This approach is very interesting. It has been conducted in a recent article by Erhart (2023) who uses the LISS panel data in the Netherlands. The design is, of course, very different from what we suggest as the author uses time series fixed effects models to examine, among other things, how timing (time periods after change in representation) shapes citizens’ political support—while we use a regression discontinuity design and permutation tests. We believe that our approach is very different, but panel data are insightful, and we now discuss this study by Erhart (2023). See this new bit from the literature review:

“Attempts to isolate the causal effect of winning/losing faces serious challenges. Moving beyond cross-sectional data, panel studies surveying respondents before and after election constitute an important step forward by focusing on within-individual differences over time [5,16,22–25]. For example, Erhart recently leveraged Dutch panel data to examine how various indicators of representation (e.g., party closeness) shape citizens’ political support [26].”

**Additional points**

The authors could make it clearer that while participation in government is one, and perhaps the most-commonly used indicator of winning or losing in politics, it is definitely not the only indicator and there is active research going one that tries to establish what makes someone (feel as) a political winner/loser and the role of media in that.

***This is a good point to which we agree. We make sure to acknowledge this point in the revised manuscript, as shown in the following paragraph from the literature review:

“In contrast to the uncertainty regarding the mechanism(s), the literature is much clearer when it comes to defining the winners and losers. Being a “winner” or a “loser” taps onto a party’s performance. Many measures can be used to capture party performance. First, one can focus on vote share, seat share and/or participation in government [17]. Second, these three sets of indicators can be either based on a given election (absolute performance) or compared to the party’s previous electoral results (relative performance) [18]. However, the focus has been put on whether a party is included or excluded from a government, that is, whether one’s party is in power or not [1]. Winners are thus voters who supported a party that ends up with cabinet members, while losers are supporters of parties ending up in the opposition. Stiers, Daoust, and Blais [19] examine citizens’ own perceptions of having won or lost an election and show that whether a voter’s party is included in government is the most important determinant of whether the voter views themselves as a winner. In other words, being in government is winning. That said, other measures like the relative vote share between the previous election and a given election is also useful, and the media’s coverage of the electoral outcomes also shape which parties are depicted as winners or losers [20,21]. At the end of the day, however, we follow the conventional wisdom, and in particular the pioneer work of Anderson et al. [1], by defining winners and losers based on whether the party they supported is included or excluded from the government.”

The data is consistent (cf. Fig 4) with the announcement of the government increasing satisfaction with democracy both for winners and losers in the 20 or so day after the cut-off (and in the few days before the cut-off, which is consistent with the anticipation of the news of government formation I mentioned above). I think this is an interesting result that, if confirmed by formal tests, should be mentioned.

***This is a fair point. It is possible that satisfaction with democracy increased among winners prior to the government announcement via media. Therefore, the revised manuscript now includes an additional empirical analysis, which estimates separate RDDs for each day within +/- 30 days around the actual cutoff. The findings are presented in the newly added Fig 5 in the revised version of the manuscript. See below:

“The upper left panel in Fig 4 showing that satisfaction with democracy among winners seems to increase some 20 days before the cut-off could be consistent with reasonable hypothesis that voters may anticipate the news about the government formation even before they are published by media in response to which their satisfaction with democracy increases. This would be problematic for our identification strategy: respondents could find out about their winner/loser status slightly before the status become known due to rumors. In fact, it is also plausible that respondents found out about their electoral status slightly after the incoming government is publicly announced via media because people do not always follow the news closely enough. We test these possibilities by varying the cutoffs along the time dimension. For each day until 30 days before and 30 days after the actual cutoff, we estimate separate RDD’s. One caveat to this type of placebo analysis is that we expect to see about 3 false positives per plot because we estimate 95% confidence intervals and estimate 60 models. Fig 5 shows the results for the pooled models (while S-D1 Fig in Appendix D shows the effects for each separate election).

The findings are not consistent with the anticipation hypothesis. The left panel of Fig 5 shows the results, and one would expect from the anticipation mechanism that winners should become more satisfied with the way democracy works once they find out about their electoral status. There are only two positive and significant effects (out of 60 estimates) in the left panel (winners only): One five days before the actual cutoff and one fifteen days after. Both of these effects could be false positive effects. The right panel illustrates the effects for the electoral losers. Electoral losers should become less satisfied once they learn their electoral status. Here, we therefore expect a negative effect. There is one significant negative effect in the plot at ten days after the actual cutoff date. Overall, across both plots there are three effects (out of 120 estimates) that are significant and in the expected direction of the anticipation mechanism. However, the dates do not overlap for winners and losers which goes against the anticipation hypothesis. Indeed, we would expect both winners and losers to learn about their electoral status at about the same day (not 15 days apart—from the positive effect among winners at day -5 to the negative effect among losers at day +10).”

We are grateful for the reviewer’s insightful observation, which prompted us to further examine the data and enhance the robustness of our findings.

The conclusions of the article can do more to unpack the significance of the results and consider the limitations imposed by the selection of countries, elections, and government formation outcomes (and shorten the summary of findings in that section).

***This is a fair point. We revised the last paragraph of the conclusion so that it highlights the significance of our findings, notably because they do not seem to replicate one of the most robust findings in political science (i.e., the winner-loser gap in citizens’ satisfaction with democracy), and made sure to mention the limitation due to the samples:

“Our results do not reveal any satisfaction boost among winners, nor satisfaction decay among losers. Hence, we do not replicate what is systematically found in social science research [e.g., 1–5]. This is puzzling, especially given the robustness and consistency of our key finding from three different contexts and several alternative methodological choices.

“One of the key implications of our research is that the very act of parties entering government which translates into one’s winner/loser status according to the literature seems to be a mere proxy in explaining the winner-loser gap. In other words, party government status does not seem to explain why winners tend to be more satisfied with democracy than losers. However, our findings are limited to a set of well-established democracy using proportional representation. Further research should aim to expand cases study outside well-established democracies to assess the external validity of our findings, and should also aim at testing contextual effects related to whether the winners supported a major or junior coalition member of the government, as well as levels of polarisation of a party systems.”

**Reviewer #2**

The manuscript has employed an interesting empirical and methodological strategy to strengthen the causal evaluation of the theory of the winner-loser gap, yielding an unexpected result. However, I believe that the manuscript could be enhanced both in terms of theoretical framing and conclusion formulation, as well as in its empirical analyses. Firstly, in my understanding, the quasi-experimental approach employed by the authors should be regarded as a tool to ascertain which of the two mechanisms proposed to explain the winner-loser gap—emotional or utility-based—is more appropriate or robust, rather than testing the theory as a whole. The manuscript itself presents reasoning akin to this when it criticizes studies based on observational data: “Fundamentally, however, these models cannot disentangle the effects of the positive emotional effect of having voted for a widely supported party from the effect of having voted for a party that controls the government” (rows 136–8).

Therefore, it could be argued that even if the difference in the degree of satisfaction with democracy between winners and losers is attributed to differences in party support in the electorate (the omitted variable that would cause bias, according to the authors), the theory itself remains valid. However, the specific mechanism underlying it is linked to the positive and negative emotions engendered by being in the majority or minority in society.

In this sense, given that the manuscript’s methodology allows for circumventing the previous knowledge of the winner-loser position, I believe that the authors actually test the occurrence of the utility mechanism, which is related to political system outcomes. Therefore, I suggest that the authors present data related to party and electorate polarization in the analyzed elections. If these statistics indicate weak polarization, the null results found could be interpreted as demonstrating that, in the cases examined, weak party and electorate polarization explain the null result, suggesting that it may be the more important mechanism for explaining the occurrence of the winner-loser gap.

***These are very good points. First, it made us realize that we were not clear enough regarding the mechanism at play, especially given that there can be two different emotion-based and one utility-based mechanisms. We now include a discussion on the mechanism that could be at play and which one we can properly isolate. See below:

“In taking up this research design, we aim to separate two distinct mechanisms for the winner-loser gaps. The first mechanism, which we have noted above is sometimes regarded as an “emotional” mechanism, is that in which voters who vote for more popular parties are more satisfied precisely because that party is more popular. Being onside with a large share of other citizens and perhaps knowing that they share one’s views may be sufficient to generate more satisfaction with democracy. This can be labelled as a “popularity mechanism”. Note that this emotion-based mechanism is different than the hypothesis suggesting that people are more satisfied with democracy when they feel that they “won” the election [16].

But because parties which earn a greater share of the vote are, *ceteris paribus*, more likely to be in government, this may conflate with the status of having voted for a party included in the government, which is a different mechanism. This second mechanism involves having the party you support actually be in government. Having one’s preferred party in government can confer both symbolic benefits and material ones, and both can be expected to increase satisfaction with the democracy. This can be called a “government mechanism”. In this research, we aim to isolate this very mechanism.”

Second, it would have been useful to test whether differences would emerge across winners and losers whose party vary in terms of their vote shares. However, providing an adequate test of this kind would require interaction terms such as “winning (ref: losing) × voting for a small party (ref: voting for a major party)”. This would be the only way to assess whether, for example, the effect of winning (that is, voting for a party that is included in the government) is heterogenous based on whether the party that the voter supported is the largest party or a junior party that is included in the coalition (Brambor et al. 2006). While this would be insightful to investigate, the data do not include enough observations to have the statistical power to conduct such interaction effects because we need variance both for the winner/loser status as well as the size of the party.

All in all, our design allows to better isolate the “government mechanism” but we cannot analyze the popularity mechanism. In the revised conclusion, we now mention that further research should strive to include more cases to test different contextual effects including the size of the party that the winners supported (i.e., is the effect the same for winners who supported the most popular party compared to junior coalition partners?):

“Further research should aim to expand cases study outside well-established democracies, especially given the larger size of the gap in such context [7], to further assess our findings in a variety of contexts and eventually test contextual effects related the quality of the democratic regimes, but whether the winners supported a major or junior coalition member of the government, as well as levels of polarisation.”

Third, the level of polarization might be link with the size of the winner-loser gap, but unfortunately, the adequate way to formally test this hypothesis would be to have enough cases that vary on their levels of polarisation, which we cannot do. We now acknowledge that our samples of democracies include well established consensual democracies (p. 5) which are less polarized than majoritarian democracies. However, our case studies are not that much less polarized compared to other well established democracies and the difference should not be overstated. While we cannot provide a systematic and comparable measure of polarization with the datasets that we use, data from Bernaerts et al. (2023: Figure 2) provides useful information. For example, out of 36 countries studied, only 13 were more polarized than the Netherlands on their idea-based polarisation. Iceland was more polarised than countries like Japan, Germany, Czech Republic, Latvia, etc. on their measure of identity-based polarisation. Norway’s level of idea-based polarisation was similar to countries like Portugal, Lithuania, etc. That said, thanks to the reviewer’s comment, we now fully acknowledge the limitation of our sample and their aggregate-level characteristics (including their electoral system and levels of polarisation) and mention explicitly that further research should strive to examine more thoroughly the role of polarisation with the appropriate statistical power.

To finish, one minor point. In rows 102–3, the manuscript says, “However, losers are systematically less likely to express political support compared to losers.” I think the second “losers” should be replaced by "winners.”

***We thank R2 for spotting this error which is now corrected.

**Reviewer #3**

**General assessment**

The paper addresses whether the winner-loser status explains satisfaction with democracy. By applying a regression discontinuity design to data from three elections—Norway 1997, the Netherlands 2010, and Iceland 2017—the authors’ main finding do not confirm the theoretical expectations. In other words, winners do not show higher levels of satisfaction with democracy, and losers are not less satisfied with democracy. Although limited to three cases, the causal inference design allows for their main claim: the winner-loser gap does not explain satisfaction with democracy, challenging decades of knowledge regarding the topic.

Whereas the paper’s main strength is establishing a causal relation between the winner-loser status and satisfaction with democracy, its weakness might be the impossibility of external validity. On the one hand, the authors address the question through a careful research design that considers the assumptions of RDD and assigns robustness to their findings through standard tests. On the other hand, it is hard to claim that such findings are valid for other cases rather than those analyzed.

***We thank Reviewer 3 for their positive review and agree about the limitation of our paper regarding the external validity of our sample. We address this point below.

**Major issues**

The paper has no significant issues.

**Minor issues**

The way in which the authors present their conclusion could be done more carefully. Both in the introduction (lines 97-98) and conclusion (lines 303-306 and 310-311), the authors state their conclusion in a way that might make it seem as if previous literature is misleading - which is not accurate. Although prior tests usually rely on probabilistic research designs (therefore, establishing a correlation between variables) using the winner-loser gap as one of the predictors of various dependent variables outcomes (satisfaction with democracy, trust in political institutions, etc.), the study of three cases alone does not falsify all previous research. The authors are not explicitly arguing this, but the way that they present it – that “party government status does not seem to explain why winners tend to be more satisfied with democracy than losers” – sounds like the findings are generalizable.

***This is a fair point. Based on the reviewer’s comment, we made sure to tone down our claims. First, we are now much more explicit when discussing the limitation of the external validity of our sample. For example, we revised the bits mentioned by the reviewers as follow:

[From the introduction] “One implication is that even though the literature attributes the differences in satisfaction among winners and losers to the parties’ access to the government, our findings make it look unlikely that this is the whole story in our sample of well-established consensual democracies (Norway, Netherlands and Iceland). The causal mechanism behind the winner-loser gap appears to be more complex and more research should address this puzzle in several ways that we discuss in the conclusion.”

[From the conclusion] “In other words, party government status does *not* seem to explain why winners tend to be more satisfied with democracy than losers. However, our findings are limited to a set of well-established democracy using proportional representation. Further research should aim to expand cases study outside well-established democracies to assess the external validity of our findings, and should also aim at testing contextual effects related to whether the winners supported a major or junior coalition member of the government, as well as levels of polarisation of a party systems.”

See also our response to the next point for more details regarding the external validity.

Approaching the issue through causal inference techniques is undoubtedly an important step. However, do the conclusions apply beyond these cases? Is there external validity? The findings bring some theoretical implications because theoretical expectations are not confirmed. Yet, the authors should be more cautious in presenting their argument, clarifying that the conclusion only applies to the cases under scrutiny.

***As mentioned above, we agree with this comment. Based on R3’s comment, we now better highlight this limitation in the revised iteration of the manuscript.

-In the introduction, we clarified “in well-established democracies” when referring to our main conclusion.

-In the method section, we added the following:

“The external validity of our case studies is constrained by the fact that the three countries included are not representative of most democracies in the world. Indeed, they are well established democracies using proportional representation, which has been shown to reduce the winner-loser gap [34]. That said, even democracies of highest quality still display a winner-loser gap in citizens’ political support [35]. Indeed, Erhart mentions that “The winner-loser gap persists even in a consolidated, well-functioning consensus democracy like the Netherlands.” [26] However, given the characteristics of the countries included in our samples, we do not extrapolate our findings to democracies in general, but rather to the three case studies that we could include.”

-We conclude the paper by calling for further research that would improve the external validity of our findings:

“However, our findings are limited to a set of well-established democracy using proportional representation. Further research should aim to expand cases study outside well-established democracies to assess the external validity of our findings, and should also aim at testing contextual effects related to whether the winners supported a major or junior coalition member of the government, as well as levels of polarisation of a party systems.”

The minor issue mentioned above is the most crucial one. Below, I point to a few less substantial but important things to address.

The article needs to be revised for typos and misplaced sentences. A few sentences appear in the main text but should be footnotes or endnotes (lines 197-200 and 212-214, for instance).

***We agree with R3 that it would have been useful to put some information as footnotes, but PLOS ONE does not allow footnotes, so we moved them in the core of the text. Moreover, we asked a native English-speaking colleague to read the entire manuscript to correct as many typos as possible.

Regarding how authors present their findings, I suggest bringing Table 4 from the Appendix to the text.

*** Thank you for the suggestion. Originally, we did not want to overwhelm the reader by including the table, as the corresponding information is presented in Fig 4. However, we have now added this table to provide greater clarity to our analysis. The previous Table 4 from Appendix C is now included in the main part of the manuscript as Table 1, along with its interpretation.

Figure 4 presents the results. I believe the authors are plotting polynomial regressions, which is a valid method. However, I wonder if a simple linear fit would be better for visualizing continuity assumptions. Although the authors do not confirm expectations, they find significant results contrary to the expectations for winners. Therefore, the running variable discontinuously jumps at the cutoff—even if it jumps downwards. Would the jump occur in the absence of the treatment?

***We thank the reviewer for pointing out that our approach was not clear and added the following to the manuscript: “We estimate satisfaction with democracy at and around the cutoff following the current standard approach via local polynomial regression with a first order polynomial to avoid overfitting [44,45].” We do not use global linear regression as this is not common in current applications of RDDs, but we would be happy to implement this alternative specification if a methodological piece that was under our radar shows that it is adequate for the application of a RDD.

Still related to Figure 4, the authors could centralize the cutoff on the plot. The X-axis should have the same number of days on each side of the cutoff.

***We do not centralize the cutoff in Fig 4 to emphasize that the bandwidths are different either side of the cutoff. The bandwidth sizes depend on the number of respondents. We follow the standard approach from the literature to algorithmically determine the bandwidths on both sides of the cutoff separately which is optimal in terms of minimizing the bias-variance tradeoff [41].

Overall, this is a fascinating and promising paper that applies cutting-edge methods to a classic question in public opinion studies. Therefore, my recommendation is to revise minor issues and resubmit.

** We are grateful to R3 for their positive and useful review.
